# Towards clinical application of 7T TOF angiography

**DOI:** 10.1186/1532-429X-14-S1-W72

**Published:** 2012-02-01

**Authors:** Sebastian Schmitter, Edward J Auerbach, Gregor Adriany, Kamil Ugurbil, Pierre-Francois Van de Moortele

**Affiliations:** 1 University of Minnesota, Center for Magnetic Resonance Research, Minneapolis, MN, USA

## Background

As shown at 7T Time-of-Flight (TOF) angiography significantly benefits from ultra high field (UHF) [1,2], because of shorter T1 relaxation constant and increased SNR. However, significant challenges have to be overcome before using 7T TOF in clinical applications: I) RF induced specific absorption rate (SAR) substantially increases at UHF, preventing the use of standard RF pulses (venous Saturation (SAT), Magnetization Transfer (MT)) to improve TOF contrast. II) Severe transmit B1 (B1+) heterogeneity at 7T impairs contrast homogeneity.

## Methods

In this work we approached both challenges with complementary strategies:

1) SAR reduction with VERSE [3,4] for excitation and travelling venous SAT pulses. The max amplitude of VERSE RF pulses was set to a fraction κEXC (or κSAT) of the max amplitude of initial excitation (or SAT) pulse. 2) Sparse application of MT [5] (10% of the centered k-space lines in phase and slab direction [6]).

3) Improved homogeneity of RF excitation profile with B1+ shimming (16 transmit channels), using fast multi channel B1+ calibration [7]. The standard deviation divided by the mean (std/mean) of B1+ was minimized over the center slices of 3 TOF slabs using nonlinear optimization algorithms.

## Results

**Figure 1:**
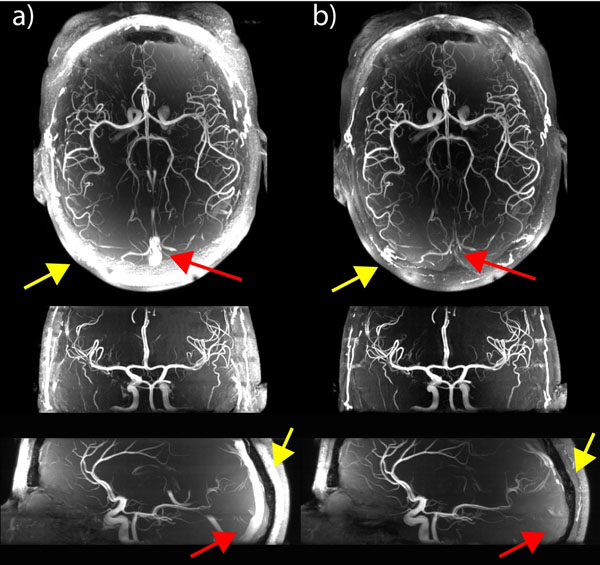
MIP images of the original TOF (a) without VERSE, venous SAT or MT and the modified TOF sequence (b) with venous SAT and with MT acquired at 7 Tesla. Successful suppression of venous signal (red arrows), significant fat signal supression (yellow arrows) as well as improved overall background suppression is visible in b). Images were not corrected for receive profile for better visualization of the individual effects.

1+2) Fig.1 shows maximum intensity projections (MIPs) for a) the original sequence with κEXC =100% (no VERSE), without SAT and without MT, and b) our modified sequence with κEXC=50%, with venous SAT (κSAT=25%) and sparse MT. Quantitative results: vessel to tissue contrast improved by >50%, venous signal suppressed by ~90%. Strong fat signal suppression observed within the excitation slab results from the large spatial chemical shift of VERSE-SAT pulse. SAR values were, for a) and b), 17% and 69% of maximum SAR limit, respectively. By comparison, using standard excitation, SAT and MT RF pulses (no VERSE/no sparse RF) would amount 319% of SAR limit.

**Figure 2:**
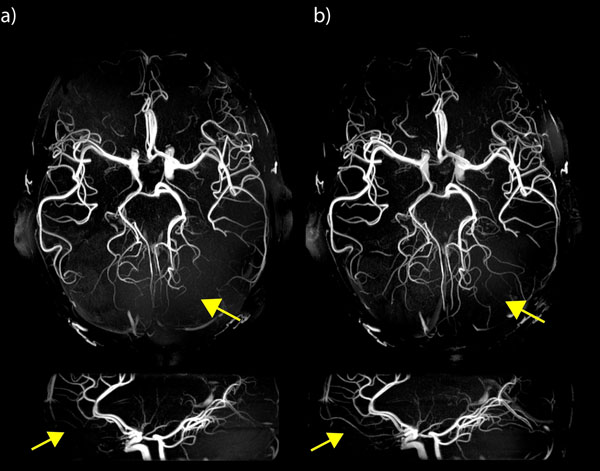
7 Tesla TOF datasets with a) circular polarized RF mode used for excitation and venous saturation, b) CP mode used for saturation and B1 phase shimming for optimized homogeneity used for excitation pulses. Smaller vessels become visible especially in the brain periphery (yellow arrows). Estimation of the spatial receive profile variations removed from both datasets for better visibility of smaller vessels.

3) Homogeneity strongly improved with B1+ shimming (Fig.2), with std/mean reduced by a factor 2.2. Smaller vessels in the brain periphery show higher contrast or become visible (see arrows). In general, however, spatial homogeneity optimization reduces B1+ efficiency, thus increases SAR. For example, for the excitation pulse SAR with B1 shimming was 4.7 times the value without B1 shimming, however this pulse represents only a fraction of SAR.

## Conclusions

We demonstrate, with appropriate RF pulse design, substantial gains in contrast and B1+ homogeneity for TOF angiography at 7T. These results strongly support the potential of expanding non-contrast enhanced angiography at 7T towards clinical investigations.

## Funding

ACKNOWLEDGEMENT: DFG: SCHM 2677/1, NIH P41 RR008079, P30 NS057091, R01 EB000331, NIH R21 EB009133; Keck Foundation.

## References

[B1] Kang MRM 2009 61 136 144

[B2] Zwanenburg JMRI 2008 28 1519 1526 10.1002/jmri.21591 19025959

[B3] Conolly J Magn Reson 1988 78 440 447

[B4] Schmitter MRM 2010 64 1447 1453

[B5] Parker MRM 1995 34 283 286 10.1002/mrm.1910340221 7476089

[B6] Schmitter MRM in press

[B7] Van de Moortele Proc. ISMRM 2007 1676

